# Cutaneous Hypersensitivity as an Indicator of Visceral Inflammation *via* C-Nociceptor Axon Bifurcation

**DOI:** 10.1007/s12264-020-00577-5

**Published:** 2020-09-09

**Authors:** Yehong Fang, Shu Han, Xiaoxue Li, Yikuan Xie, Bing Zhu, Xinyan Gao, Chao Ma

**Affiliations:** 1 Institute of Basic Medical Sciences, Department of Human Anatomy, Histology and Embryology, Neuroscience Center, Chinese Academy of Medical Sciences, School of Basic Medicine, Peking Union Medical College, Beijing, 100005 China; 2 Department of Physiology, Institute of Acupuncture and Moxibustion, China Academy of Chinese Medical Sciences, Beijing, 100700 China; 3 Joint Laboratory of Anesthesia and Pain, Peking Union Medical College, Beijing, 100730 China

**Keywords:** Visceral inflammation, Cutaneous hypersensitivity, Axon bifurcation, C-nociceptor

## Abstract

Pain on the body surface can accompany disorders in the deep tissue or internal organs. However, the anatomical and physiological mechanisms are obscure. Here, we provided direct evidence of axon bifurcation in primary C-nociceptive neurons that innervate both the skin and a visceral organ. Double-labeled dorsal root ganglion (DRG) neurons and Evans blue extravasation were observed in 3 types of chemically-induced visceral inflammation (colitis, urocystitis, and acute gastritis) rat models. In the colitis model, mechanical hypersensitivity and spontaneous activity were recorded *in vivo* from double-labeled C-nociceptive neurons in S1 or L6 DRGs. These neurons showed significantly enhanced responses to both somatic stimulation and colorectal distension. Our findings suggest that the branching of C-nociceptor axons contribute to cutaneous hypersensitivity in visceral inflammation. Cutaneous hypersensitivity on certain locations of the body surface might serve as an indicator of pathological conditions in the corresponding visceral organ.

## Introduction

Visceral pain, involving thoracic, abdominal, and pelvic organs, is the most common type of chronic pain nowadays [1–3]. However, the poor understanding of its pathogenesis makes it difficult to manage. It is well recognized that visceral innervation is complex. Organs in the thoracic and abdominal cavities may be innervated by both the vagal and spinal nerves with central terminals in the brainstem and spinal cord, respectively. Meanwhile, some pelvic organs are innervated by the pelvic nerves, which terminate in the lumbosacral spinal cord [4, 5]. Unlike cutaneous pain that is well localized, visceral pain is diffuse and often referred to a distal superficial location [4, 6]. Therefore, it is necessary to find an indicator to help detect and locate visceral pain in order to treat visceral diseases better.

Previous studies indicated that virtually all second-order spinal dorsal horn neurons, like wide dynamic range neurons, receive both visceral and convergent somatic (non-visceral) input [7, 8]. Such viscerosomatic convergence onto second-order spinal neurons has long been considered the underlying structural basis for pain in somatic sites. *In vivo* animal studies, including single-fiber recordings and spinal extracellular recordings, have revealed that visceral nociceptors (mainly mechanoreceptors) can be sensitized by inflammation and the mediated pain can be relieved by nociceptive stimulation of the skin [9, 10]. However, these findings do not negate the possibility that DRG neurons also play a role in visceral inflammation and the referred visceral sensation.

The bifurcation and sensory convergence of DRG neurons have been described in various studies [11–13]. Electrophysiological recordings from DRG neurons showed that some responded to both somatic and visceral stimulation [4], suggesting the bifurcation of primary sensory axons. In this study, we provide direct evidence that the double-labeled DRG neurons innervate both the visceral organs and skin, and used *in vivo* electrophysiology and Evans blue extravasation to confirm that cutaneous hypersensitivity can be used as an indicator of visceral inflammation *via* C-nociceptive sensory axon bifurcation.

## Methods

### Animal

Adult female Sprague-Dawley rats (specific pathogen free, 180 g–220 g, provided by the National Institutes for Food and Drug Control, China) were used. Room temperature was maintained at 23 ± 2 °C, with 50%–65% relative humidity. Rats were kept on a 12 h light/12 h dark cycle with free access to food and water. All animal experiments were approved by the Institutional Animal Care and Use Committee in the Chinese Academy of Medical Sciences, Institute of Basic Medical Sciences (Approval Number#211-2014).

### Animal Models

Colitis: The rat model (*n* = 52) of intestinal inflammation was established based on previous reports [14, 15]. Briefly, under general anesthesia (sodium pentobarbital, 50 mg/kg, i.p.; Sigma Aldrich, St. Louis, MO, USA), 100 mg/kg 2,4,6-trinitro benzene sulfonic acid (TNBS; P2297, Sigma Aldrich) dissolved in 50% ethanol were instilled into the rectum, while the control group received vehicle (50% ethanol) only. Rats showed signs of cutaneous mechanical hypersensitivity starting from day 1 after drug administration (Fig. 3J).

Urocystitis: The urocystitis model (*n* = 9) was established as previously reported [16], 300 µL of 1.5% H_2_O_2_ (Beijing Chemical Works, China) solution diluted in sterile saline was introduced into the bladder through the urethra *via* a polyethylene tube (PE-50), and kept for 30 min. Then the H_2_O_2_ solution was drained from the bladder by pressing the lower abdomen. The control group received 300 µL of sterile saline. Rats showed signs of cutaneous mechanical hypersensitivity starting from day 1 after drug administration (Fig. 3K).

Gastritis: The acute gastritis rat model (*n* = 11) was induced by intragastric administration of 1 mL of solution containing 60% ethanol plus 150 mmol/L HCL (inducer) as in a previous study [17].The control group received 1 mL sterile saline. Rats showed signs of cutaneous mechanical hypersensitivity starting from 30 min after drug administration (Fig. 3L).

All rats were examined by histological analysis. Distal colonic, vesical, and gastric tissues were harvested and stained with hematoxylin and eosin (H&E). Histopathological examination was performed with a light microscope (FV1000, Olympus, Tokyo, Japan). Tissue edema and mucosal epithelium with inflammatory cell infiltration indicated the successful establishment of rat models of visceral inflammation [18–20].

### Fluorescent Labeling

Nine rats were used in this experiment. Under anesthesia, the muscular layer of the distal colon, urinary bladder, and stomach were each injected with 20 μL of the lipophilic tracer 1,1′-dioctadecyl-3,3,3′,3′-tetrame-thylindocarbocyanine perchlorate (DiI, 200 μg/mL, Sigma Aldrich) without evident DiI leakage. After 3 days, 15 μL of the B subunit of cholera toxin Alexa Fluor 488 (CTB-488, C-34775, Molecular Probes, Eugene, USA) was injected into the skin once at multiple sides around the root of the tail and back area (thoracic segments). One week later, under pentobarbital anesthesia (50 mg/kg, i.p.), the rats were transcardially perfused with PBS followed by 4% paraformaldehyde, then DRGs in the thoracic, lumbar, and sacral segments were harvested, post-fixed for 12 h, and cryoprotected in 30% sucrose overnight. The tissue was frozen and sectioned at 13 μm on a cryostat. We cut sections at 20 μm intervals and ultimately obtained ~20 slices for each DRG. Then the sections were cover-slipped with Vectashield mounting medium with DAPI (Vector Laboratories, Burlingame, CA, USA) followed by examination under a fluorescence microscope (FV1000 and Olympus FluoView software; Olympus, Tokyo, Japan).

### Evans Blue Extravasation

Evans blue extravasation was applied after establishing the colitis (*n* = 3), urocystitis (*n* = 3), and gastritis (*n* = 3) models. Briefly, under anesthesia (sodium pentobarbital, 50 mg/kg, i.p.), rats were slowly injected with 2 mL Evans blue (1%, Sigma Aldrich) into the caudal vein and perfused transcardially with 0.1 mol/L phosphate-buffered saline 30 min later. Finally, images of corresponding local skin of each rat were collected after depilation.

### Assessment of Mechanical Pain Threshold in the Skin of Rats

Rats with colitis (*n* = 8), urocystitis (*n* = 6), and gastritis (*n* = 8) were used. Before all tests, rats were placed in a homemade square (20 cm × 10 cm) stainless-steel cage to habituate for 2 days and the hair was removed (root of the tail for colitis and urocystitis rats and thoracic back skin for gastritis rats). Habituation for 30 min was allowed before each test. The average threshold at –2 days and –1 day was defined as baseline. A calibrated electronic von Frey filament (Electronic von Frey 2390-5 Aesthesiometer; IITC Life Science, Woodland Hills, CA, USA) was applied to measure the mechanical threshold of each rat at different time points. The average of three repeated measurements was taken as the final threshold.

### *In Vivo* Electrophysiological Recording

*In vivo* extracellular electrophysiological recordings of double-labeled (labeled by both DiI and CTB-488) L6 or S1 DRG neurons were performed in both normal (*n* = 10) and colitis (*n* = 15) rats. Specific surgical details and recording procedures were as previously reported [21, 22]. Briefly, rats were anesthetized with pentobarbital sodium (initial dose of 50 mg/kg i.p. followed by supplementary doses of 20 mg/kg as needed), the L6-S1 transverse process was removed, and a laminectomy was made from L5 to S2 to expose the S1 or L6 DRG. During surgery, oxygenated artificial cerebrospinal fluid (ACSF) at 35 °C was dripped onto the surface of the DRG, and then the perineurium and epineurium were carefully removed under a dissecting microscope. After the rat was transferred to the recording platform, a pool was made by attaching the skin to a metal ring and filling it with ACSF. Action potentials were recorded extracellularly using a Multiclamp 700B amplifier (Molecular Devices, Sunnyvale, CA, USA) and Digidata 1440A (Molecular Devices, Sunnyvale, CA, USA).

To ensure that recorded neurons innervated both the inner colon and skin, we chose DiI-labeled neurons (under a fluorescence microscope) that responded to both distal colonic distension and nociceptive stimuli to the cutaneous receptive field. Axonal conduction velocity (CV) was determined by dividing the distance between the receptive field and the cell body by the latency of the action potential. A C-neuron was identified by a CV <2 m/s. The cutaneous receptive filed (RF) was identified by exploring the skin at the root of the tail using a handheld glass probe. Then various handheld stimuli were applied: cotton swab (for innocuous mechanical stimuli) and a von Frey filament with a fixed tip diameter (200 μm). For cutaneous and inner colonic stimuli, von Frey filaments were applied to the corresponding receptive field for 3 s at different forces (5 mN, 10 mN, 30 mN, and 50 mN) and the balloon in the distal colon was inflated for 10 s to a series of pressures (20 mmHg, 40 mmHg, and 60 mmHg). Spontaneous activity (SA) was defined as continuous discharge of a DRG neuron lasting for 3 min without any external stimulus. Once SA was detected, a series of stimuli, including wiping with a Q-tip, pinching with forceps, poking with an acupuncture needle, and 0.3% capsaicin, were applied to the cutaneous RF or non-RF (defined as the skin within a circle 1 cm in diameter around the RF). Each recording continued for at least another 5 min.

### Statistical Analysis

Data are presented as the mean ± SEM. Student’s *t*-test was used to test the difference between two groups. Difference among multiple groups were tested by one-way analysis of variance (ANOVA) followed by the Bonferroni *post hoc* test. A statistically significant difference was defined as a two-sided *P* value < 0.05. SPSS software (version 21.0) was used for statistical analysis.

## Results

### Dorsal Root Ganglion Neurons Innervate Both Visceral Organs and Skin

**Fig. 1:**
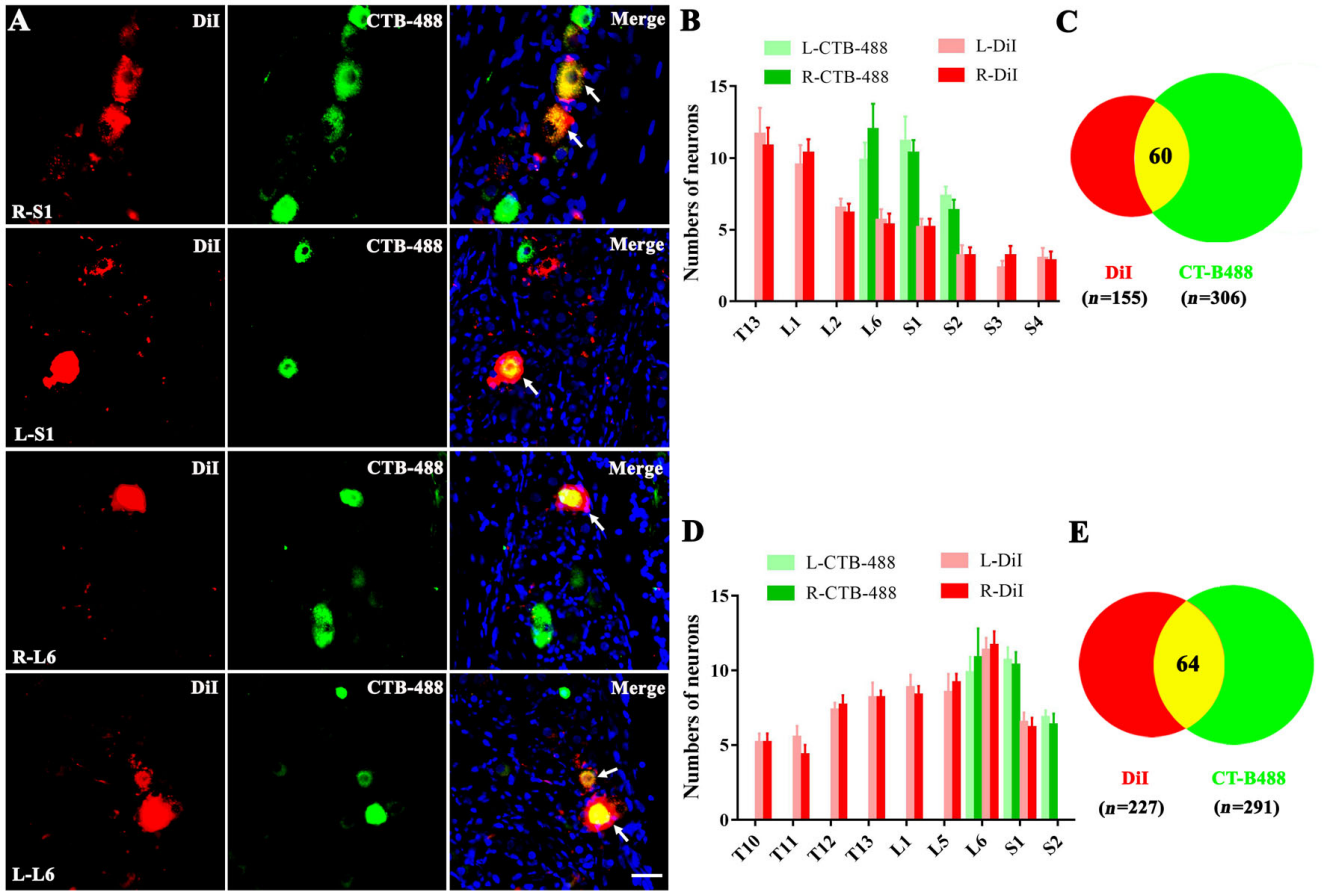
Retrogradely labeled primary sensory neurons in dorsal root ganglia (DRGs) innervating both skin and distal colon/urinary bladder. **A** Representative images of sensory neurons in bilateral L6 and S1 DRGs after injection of Dil in the distal colon and CTB-488 in the skin around the root of the tail [red, Dil-positive colon afferent fibers; green, CTB-positive skin afferent fibers; yellow, double-labeled cells (white arrows); scale bar, 25 μm; CTB-488, cholera toxin subunit B (recombinant) Alexa Fluor 488; DiI, 1,1′-dioctadecyl-3,3,3′,3′-tetrame-thylindocar bocyanine perchlorate]. **B** Bar graph of the number of neurons labeled by DiI (distal colon) and CTB-488 (skin around the root of the tail) in bilateral T13, L1, L2, L6, and S1–S4 DRGs (T, thoracic; L, lumbar; S, sacral). **C** The Venn diagram showing the total number of DiI-labeled (red), CTB-488-labeled (green), and double-labeled (yellow) neurons in all DRGs (18 DRGs from 3 rats). **D** Numbers of neurons labeled by DiI (urinary bladder) and CTB-488 (skin around the root of the tail) in bilateral T10–13, L1, L5–6, and S1–2 DRGs. **E** Venn diagram showing the total number of DiI-labeled (red), CTB-488-labeled (green), and double-labeled (yellow) neurons in all DRGs (16 DRGs from 3 rats).

**Fig. 2:**
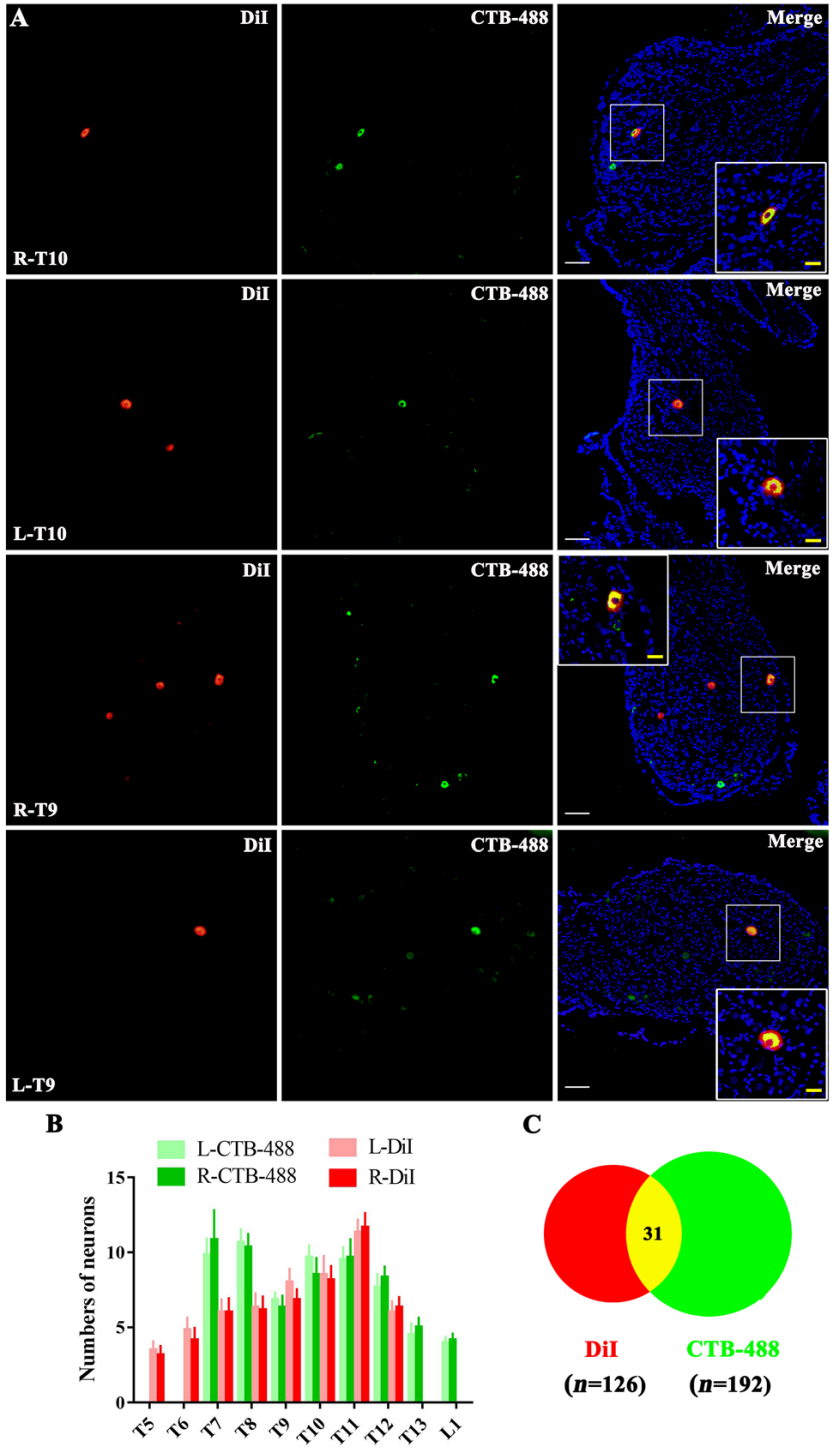
Retrogradely labeled primary sensory neurons in DRGs innervating both skin and stomach. **A** Representative images of sensory neurons in bilateral T9 and T10 DRGs after injection of Dil into the stomach and CTB-488 into the thoracic back skin. Insets are higher magnification images. Scale bars, 100 μm (white); 25 μm (yellow). **B** Counts of DRG neurons labeled by DiI (stomach) and CTB-488 (back skin of thoracic segments) in bilateral T5–13 and L1 DRGs. **C** Venn diagram showing the total number of DiI-labeled (red), CTB-488-labeled (green), and double-labeled (yellow) neurons in all DRGs (30 DRGs from 3 rats).

DRG neurons were double-labeled by injecting the fluorescent dye DiI into visceral organs (distal colon, urinary bladder and stomach) and CTB-488 into the corresponding skin (root of tail and thoracic back area). In colonic (*n* = 3) and urocystic (*n* = 3) rats, double-labeled neurons were limited to bilateral L6, S1, and S2 DRGs (Fig. 1A). Therefore, L6 and S1 DRGs were chosen for recording the electrophysiological activity of double-labeled neurons. DiI-labeled neurons (distal colon) were found in T13, L1–2, L6, and S1–4 DRGs, while CTB-488-labeled neurons (skin around the root of tail) were found in L6, S1, and S2 DRGs. Meanwhile, DiI-positive neurons (urinary bladder injection) were in the T10–13, L1, L5–6, and S1 DRGs. The number of labeled neurons did not significantly differ between the left and right DRGs in the two models (Fig. 1B, D). In addition, statistical analysis indicated that no more than 6 double-labeled neurons (accounting for 0.08% of ~ 7000 neurons per DRG [23]) were found in each DRG either in colonic or urocystic rats. We found a total of 60 double-labeled neurons (from 18 DRGs) in 3 rats with colonic injection and 64 double-labeled neurons (from 16 DRGs) in 3 rats with urocystic injection (Fig. 1C, E). For the stomach (*n* = 3), DiI-labeled neurons were found in the T5–T12 DRGs while CTB-488-labeled neurons were found in the T7–13 and L1 DRGs. A total of 31 double-labeled neurons were found in the T7–11 DRGs, with no more than 3 neurons in each DRG (25 DRGs from 3 rats; Fig. 2A–C).

### Colonic, Vesical, and Gastric Tissues in Rat Models

**Fig. 3:**
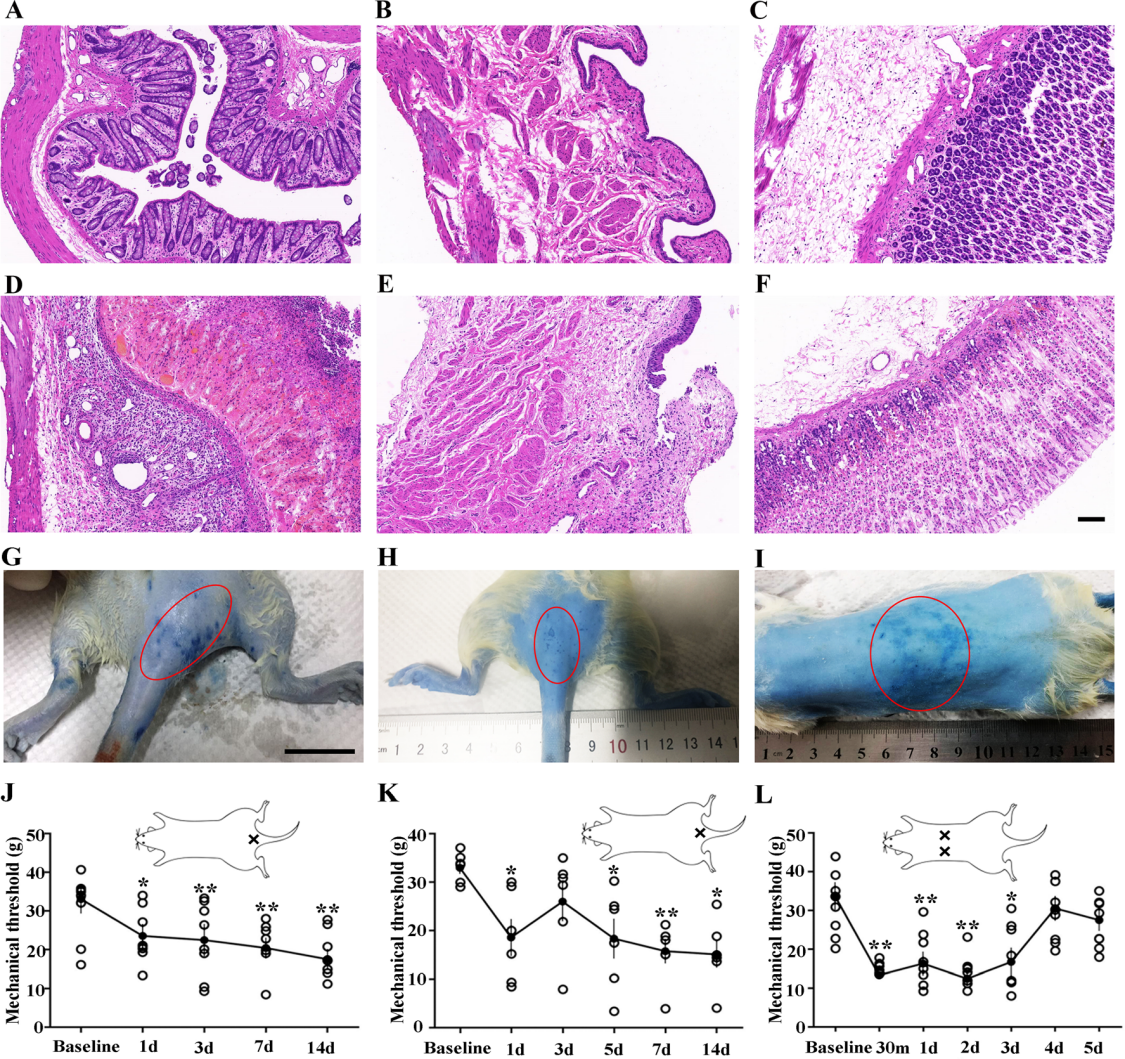
Histological changes, Evans blue extravasation and mechanical pain assessment in rat models of colitis, urocystitis, and gastritis. **A**–**C** Representative microscopic images showing H&E staining in control tissue of distal colon, urinary bladder, and stomach. **D**–**F** Representative microscopic images showing H&E staining of distal colonic, vesical, and gastric tissue sections from rat models. Scale bars in A–F, 100 μm. **G** and **H** Representative images showing Evans blue extravasation (blue dots in red circles) mainly at the root of the tail in rats with both colitis (*n* = 3) and urocystitis (*n* = 3). Scale bar in G, 2 cm. **I** Massive Evans blue extravasation (blue dots in red circles) in the thoracic back skin of rats with gastritis (*n* = 3). **J**–**L** Mechanical hyperalgesia in rats with colitis (*n* = 8), urocystitis (*n* = 6), or gastritis (*n* = 8). **J** Mechanical threshold before (baseline) and at 1–14 days after intrarectal TNBS instillation. **K** Mechanical threshold before (baseline) and at 1–14 days after transurethral delivery of H_2_O_2_ into the bladder. **L** Mechanical threshold before (baseline) and 30 min to 5 days after intragastric administration of ethanol plus HCL. *P < 0.05, **P < 0.01 *vs.* baseline, Student’s *t*-test.

After intracolonic instillation of TNBS for 3–7 days, rats were emaciated (10% loss of body weight)and mortality was high (50%, 26 out of 52 survived) as reported previously [14]. Compared to H&E-staining of control tissue (Fig. 3A–C), an absence of part of the mucosal layer and inflammatory cell infiltration in the mucosal epithelium and muscular layer were observed in colonic tissue (Fig. 3D), while edema and inflammatory cell infiltration were observed in vesical tissue (Fig. 3E). In addition, intermittent rupture of the muscular layer and exuviation of urothelium were observed. Similar changes occurred in H&E-stained gastric tissue (Fig. 3F). The above results indicated that models of colitis, urocystitis, and gastritis had been successfully established.

### Cutaneous Mechanical Hypersensitivity and Inflammation in the Model Rats

Cutaneous mechanical hypersensitivity and neuroinflammation have been described in animals with visceral pain [24, 25]. To confirm the location of sensitized areas on the skin, extravasation of Evans blue was performed. As Fig. 3G showed, Blue dots were observed in the skin at the root of the tail in rats with colitis, indicating the appearance of neuroinflammation in this site. And in rats with urocystitis, Evans blue was exuded at almost the same site around the root of the tail (Fig. 3H). Rats with gastritis provided the strongest evidence, with massive Evans blue extravasation on the thoracic back skin (Fig. 3I).

Mechanical hypersensitivity was assessed in the model rats (colitis, *n* = 8; urocystitis, *n* = 6; gastritis, *n* = 8). Compared to baseline, the cutaneous mechanical pain threshold of all the model rats declined significantly as early as 30 min after intervention and remained for up to 14 days in rats with colitis and urocystitis, and 3 days in those with gastritis (Fig. 3J–L).

**Fig. 4:**
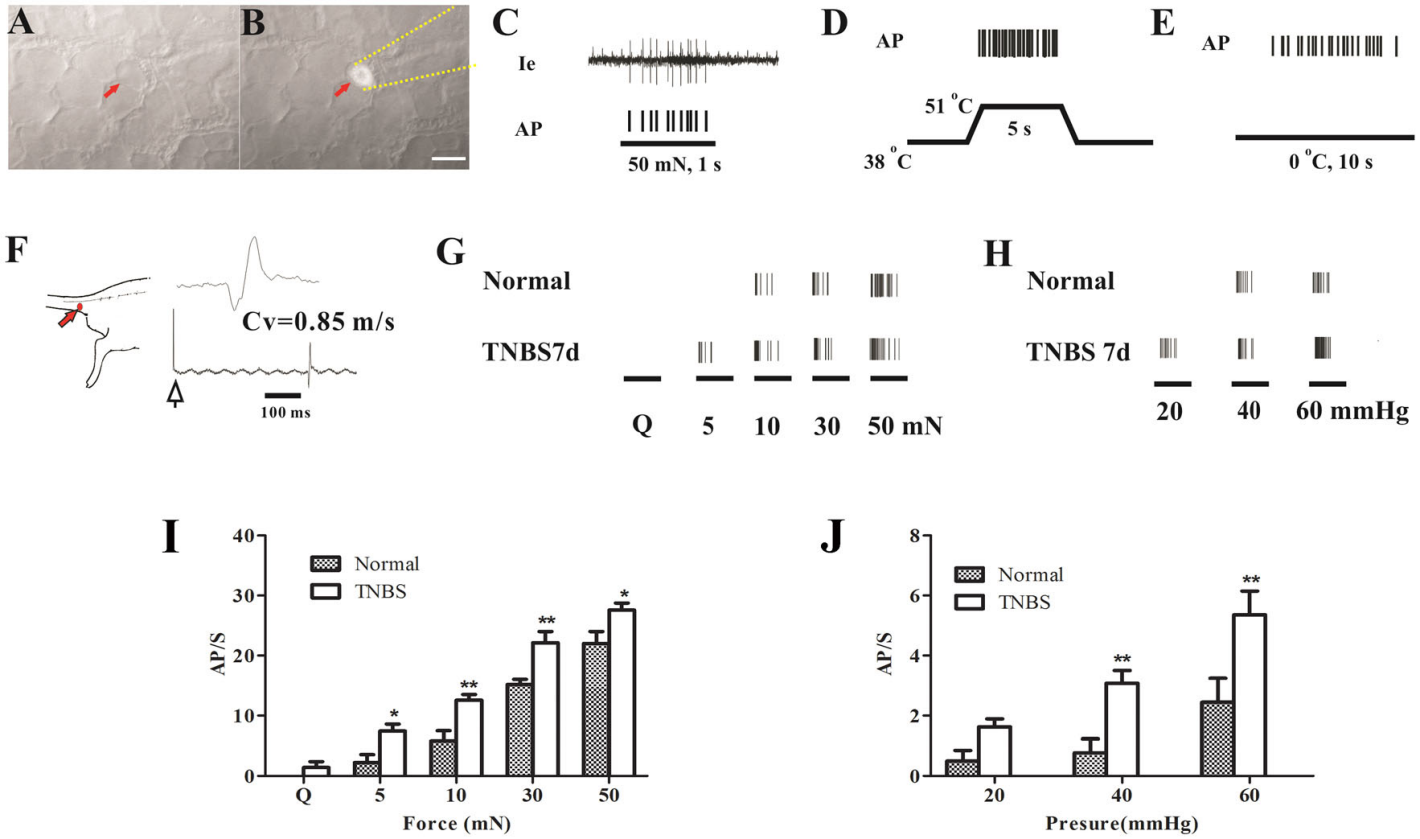
Mechanical threshold of double-labeled C-nociceptive neurons in S1 and L6 DRGs from control and TNBS-treated rats. **A** Bright-field image of the surface of an S1 DRG; red arrow indicates a small neuron. **B** Fluorescence image of the same cell under recording by an extracellular glass electrode (orange dotted line). Scale bar for B and A, 20 μm. **C** Typical response of a double-labeled small neuron to 50 mN mechanical stimulation. Action potentials (APs) in the original recording (Ie) are indicated by corresponding tick marks below. **D** and **E** This neuron also responds to nociceptive warm (51 °C) and cold (0 °C) stimulation. **F**. Measurement of conduction velocity (CV) of recorded neuron by stimulation of the receptive field (RF, red arrow). **G** Responses of double-labeled C-nociceptive neurons to Q-tip wiping and von Frey filaments of several forces (5, 10 mN, 30 mN, and 50 mN) in control and TNBS-treated rats. **H** Responses of double-labeled C-nociceptive neurons to expansion of the distal colon by a balloon at several pressures (20 mmHg, 40 mmHg, and 60 mmHg) in control and TNBS-treated rats. **I** and **J**. Action potential discharge rate s (AP/s) of double-labeled C-nociceptive neurons evoked by mechanical stimulation (**I**) and balloon expansion (**J**) in control (*n* = 6) and TNBS-treated (*n* = 7) rats *P < 0.05, **P < 0.01, TNBS *vs.* normal group (**I** and **J**), one-way ANOVA with Bonferroni *post hoc* test.

*In vivo* electrophysiological recordings from the C-nociceptive neurons labeled with fluorescent dye showed RFs on both the skin and the colon. A total of 30 neurons were recorded from naïve rats (*n* = 10) and 32 from those with colitis (*n* = 15). These neurons showed significantly enhanced responses to expansion of the distal colon and mechanical, nociceptive, warm, or cold stimulation of the cutaneous RF (Fig. 4A–H), indicating both a cutaneous mechanical sensitization (5 mN, 2.20 ± 1.44 *vs.* 7.43 ± 1.13, *P* < 0.05; 10 mN, 5.80 ± 1.80 *vs.* 12.57 ± 0.98, *P* < 0.01; 30 mN, 15.20 ± 1.00 *vs.* 22.14 ± 1.89, *P* < 0.01; 50 mN, 22.00 ± 1.79 *vs.* 27.57 ± 1.20, *P* < 0.05, Fig. 4I) and visceral sensitization (40 mmHg, 3.08 ± 0.43 *vs.* 0.77 ± 0.46, *P* < 0.01; 60 mmHg, 5.36 ± 0.79 *vs.* 2.44 ± 0.80, *P* < 0.01, Fig. 4J).

### Spontaneous Activity in Colitis Model and Changes After Diverse Cutaneous Stimulation

**Fig. 5:**
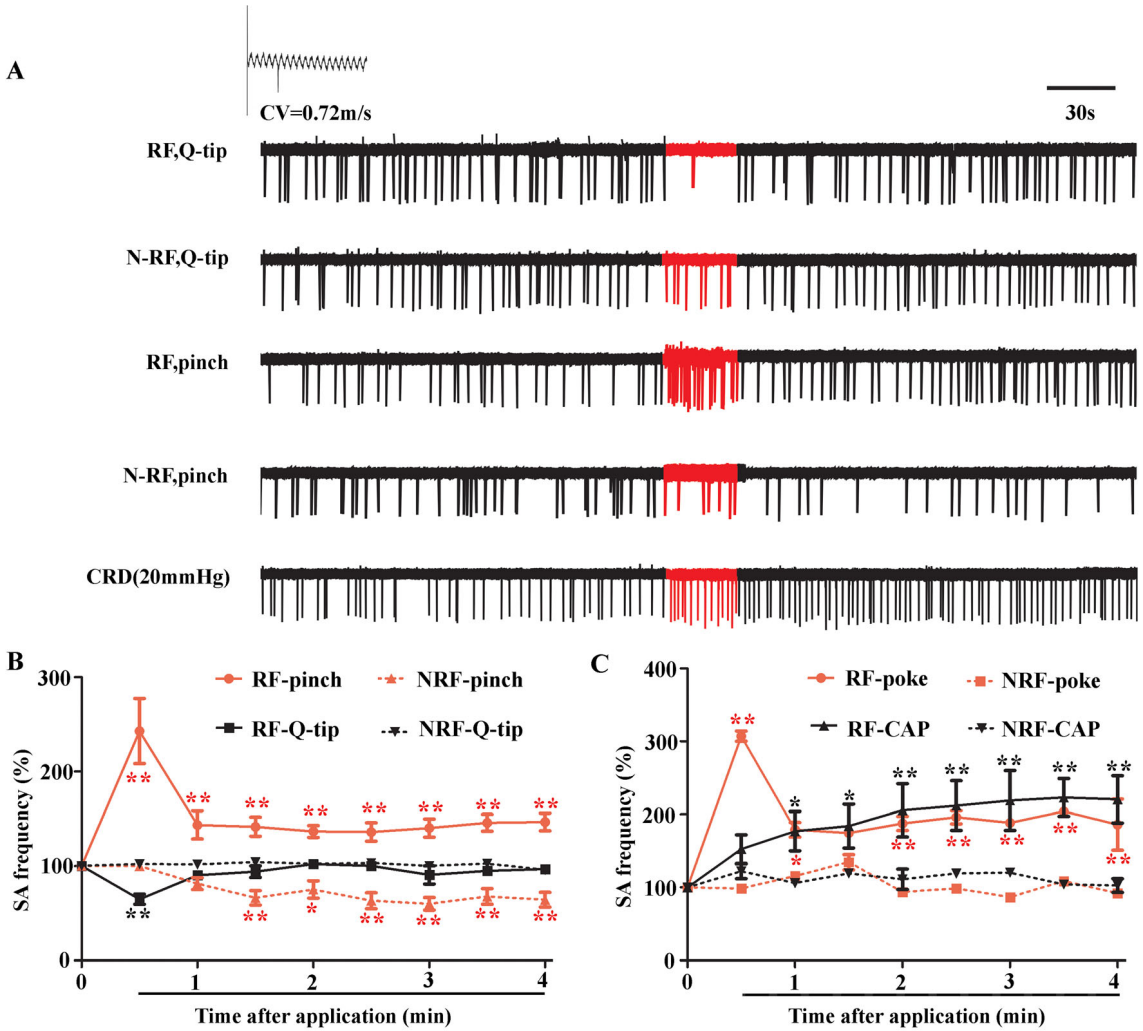
Changes in spontaneous activity (SA) after different cutaneous stimuli in rat model of colitis. **A** An example of SA recorded in one S1 DRG neuron (conduction velocity, 0.72 m/s) changing with both non-nociceptive (Q-tip) and nociceptive (pinch) mechanical stimulation applied to both receptive field (RF) and non-RF areas (red, action potentials (APs) during 30-s stimulation). The neuron responds to both cutaneous stimulation and colorectal distention (CRD). **B** Quantification of SA frequency in L6 and S1 DRG neurons after application of a Q-tip (*n* = 8) and pinch (*n* = 10) to both RF and non-RF areas. Mean SA frequency within 3 min before application is defined as 100%. *P < 0.05, **P < 0.01, after application *vs* before application, one-way ANOVA. **C** Statistics of SA frequency after applying capsaicin (CAP, *n* = 4) and poking (poke, *n* = 5) to both RF and non-RF areas. *P < 0.05, **P < 0.01, after application *vs.* before application, one-way ANOVA.

Spontaneous activity was recorded with an average frequency of 0.33 Hz (from 0.11 Hz to 0.55 Hz, data is not shown) in rats with colitis (*n* = 15), whereas no SA occurred in normal rats (*n* = 10). Application of a Q-tip to the cutaneous RF showed an immediate inhibitory effect on SA but recovered instantly once the stimulus was removed. However, a Q-tip had no effect when applied to the non-RF skin. On the other hand, SA frequency increased significantly when pinching the RF skin with forceps, but declined when pinching the non-RF skin (Fig. 5A, B). Increased SA frequency was also recorded when poking or smearing capsaicin on the RF skin, but not when poking or smearing capsaicin on the non-RF skin (Fig. 5C).

## Discussion

Visceral pain and visceral sensation are diffuse in character and often refer to other non-visceral somatic organs such as skin [4]. Thus, it is sensible to regard cutaneous hypersensitivity as an indicator of visceral pain. TNBS-induced colitis simulates, to an extent, the pathogenesis and clinical course of Crohn’s disease [26, 27], was used in our study. We found that both cutaneous hypersensitivity and neuroinflammation appeared in this model. Moreover, our results indicated that sensory axon bifurcation may be the anatomical basis for this physiological phenomenon. We then verified this finding in both urocystitis and gastritis models and got similar results. In addition, to label DRG neurons innervating both visceral organs and skin, two kinds of fluorescent dye (DiI and CTB-488) were used. Data analysis of fluorescent labeling revealed the double-labeled neurons (≤ 6 neurons per DRG) accounted for ~ 0.08% of total neurons in each DRG examined. This rate is far below the rate of 1% reported in a previous study [23], which may be due in part to the small proportion of visceral innervation among all spinal afferents.

Cutaneous hypersensitivity appears in the case of visceral pathology, indicating that visceral and cutaneous sensations communicate and may interplay. Previous studies [8, 28–32] have revealed some of the reasons for this phenomenon. One explanation is that wide dynamic range neurons in the dorsal horn (usually located in laminae I and V) receive electrical signals from both visceral organs and cutaneous stimulation, which then are integrated in the spinal cord and may in return affect cutaneous sensation by antidromic activation of the dorsal roots (dorsal root reflex theory). These reverse discharges further induce peripheral vasodilation and cause the release of substance P, calcitonin gene-related peptide, and other inflammatory substances [33]. Our Evans blue extravasation experiments further confirmed this hypothesis.

Another explanation is DRG neuron coupled activation, which is also called the cross-depolarization theory [30–32]. This means once the primary neurons innervating visceral organs are activated, the neighboring neurons (that may innervate the skin) also become excited and sensitive. Gap junctions between neurons and astrocytes can transfer information between two neurons and make possible the appearance of cutaneous sensitization in the condition of visceral pain. This possibility will be further explored in future studies.

**Fig. 6:**
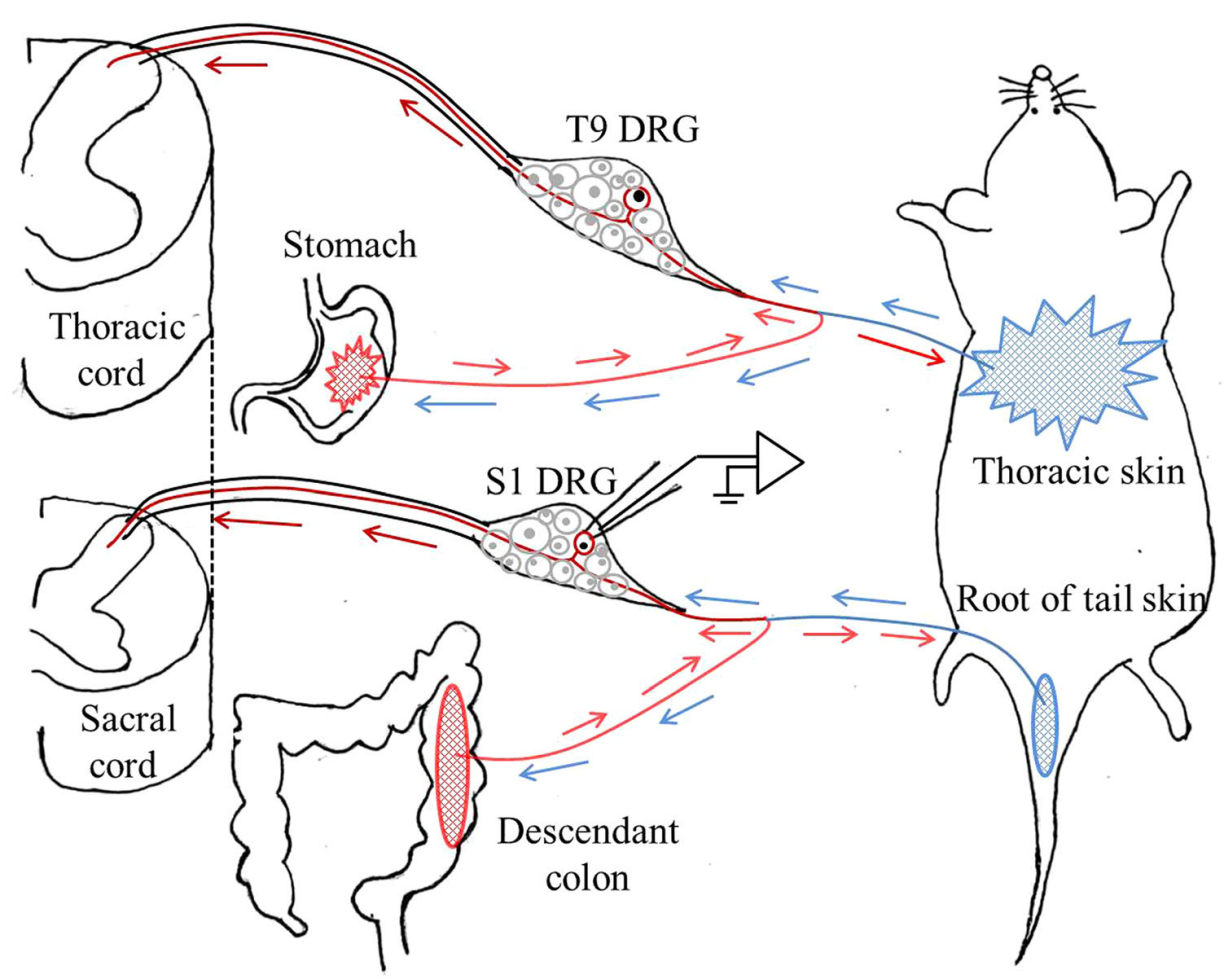
Schematic showing how axon branching of C-nociceptive neurons in dorsal root ganglia (DRGs) contributes to cutaneous hypersensitivity *via* neurogenic inflammation that occurs commonly in visceral inflammatory pain. The inflammation of visceral organs (colon and stomach) leads to discharges of C-nociceptive neurons in DRGs and further results in neurogenic inflammation and cutaneous hypersensitivity, which in turn is an indicator of visceral pain.

The bifurcation of axons in the peripheral nerve has been found in previous studies [23]. Pierau and colleagues [12] found that a number of DRG neurons respond to electrical stimulation of myelinated fibers in both the pudendal and sciatic nerves and this double-response can also be elicited when the dorsal roots are cut. These results are strong evidence that the peripheral processes of some ganglion cells dichotomize into different nerves innervating their respective target organs. The fluorescent labeling of DRG neurons and electrophysiological recording from them in our study also revealed that one double-labeled neuron can respond to both distal colonic distension and cutaneous stimulation. The inflammation of visceral organs leads to the discharge of C-nociceptive neurons in DRGs and further results in neurogenic inflammation and cutaneous hypersensitivity, which in turn as an indicator of visceral pain (Fig. 6). Interestingly, the SA frequency of C-nociceptive neurons changed differently following nociceptive or non-nociceptive stimulation of the cutaneous RF or the skin adjacent to the RF (non-RF). To be specific, the SA was inhibited immediately by Q-tip wiping on the cutaneous RF while unreactive to touch of the non-RF area. This result supports the hypothesis that innocuous afferent input from touch of the skin may alleviate acute pain as described in previous studies [34, 35]. By comparison, the SA was accelerated and maintained for several minutes when the RF received nociceptive stimulation (pinch and poke) while reduced with stimulation of the non-RF. Unexpectedly, poking and capsaicin applied to the non-RF area failed to induce any significant change of SA. One explanation for these results might be that visceral pain is inhibited only if a large number of C-nociceptors in the non-RF skin are activated and adequate afferent signals are delivered to the spinal cord. This hypothesis will be further explored in future. Taken together, our findings suggest that axon branching of C-nociceptors contributes to cutaneous hypersensitivity in visceral inflammation, and cutaneous hypersensitivity at certain locations on the body surface might serve as an indicator of pathological conditions in the corresponding visceral organ.

## References

[1] Ananthakrishnan AN (2015). Epidemiology and risk factors for IBD. Nat Rev Gastroenterol Hepatol.

[2] Elsenbruch S, Hauser W, Janig W (2015). Visceral pain. Schmerz.

[3] Durazzo M, Gargiulo G, Pellicano R (2018). Non-cardiac chest pain: a 2018 update. Minerva Cardioangiol.

[4] Gebhart GF, Bielefeldt K (2016). Physiology of visceral pain. Compr Physiol.

[5] Cervero F. Sensory innervation of the viscera: peripheral basis of visceral pain. Physiological reviews 1994, 74. 10.1152/physrev.1994.74.1.95 8295936

[6] Cervero F (1995). Visceral pain: mechanisms of peripheral and central sensitization. Ann Med.

[7] Zain M, Bonin RP. Alterations in evoked and spontaneous activity of dorsal horn wide dynamic range neurons in pathological pain: A systematic review and analysis. Pain 2019. 10.1097/j.pain.0000000000001632 31149976

[8] Ness TJ, Gebhart GF. Interactions between visceral and cutaneous nociception in the rat. I. Noxious cutaneous stimuli inhibit visceral nociceptive neurons and reflexes. J Neurophysiol 1991, 66: 20–28. 10.1152/jn.1991.66.1.20 1919667

[9] Zhao JJ, Rong PJ, Shi L, Ben H, Zhu B (2016). Somato stimulation and acupuncture therapy. Chin J Integr Med.

[10] Zhang R, Lao L, Ren K, Berman BM (2014). Mechanisms of acupuncture-electroacupuncture on persistent pain. Anesthesiology.

[11] Armijo-Weingart L, Gallo G (2017). It takes a village to raise a branch: Cellular mechanisms of the initiation of axon collateral branches. Mol Cell Neurosci.

[12] Pierau FK, Taylor DC, Abel W, Friedrich B (1982). Dichotomizing peripheral fibres revealed by intracellular recording from rat sensory neurones. Neurosci Lett.

[13] Christianson JA, Liang R, Ustinova EE, Davis BM, Fraser MO, Pezzone MA (2007). Convergence of bladder and colon sensory innervation occurs at the primary afferent level. Pain.

[14] Antoniou E, Margonis GA, Angelou A, Pikouli A, Argiri P, Karavokyros I (2016). The TNBS-induced colitis animal model: An overview. Ann Med Surg (Lond).

[15] Zheng H, Chen M, Li Y, Wang Y, Wei L, Liao Z (2017). Modulation of gut microbiome composition and function in experimental colitis treated with Sulfasalazine. Front Microbiol.

[16] Dogishi K, Okamoto K, Majima T, Konishi-Shiotsu S, Homan T, Kodera M*, et al.* A rat long-lasting cystitis model induced by intravesical injection of hydrogen peroxide. Physiol Rep 2017, 5. 10.14814/phy2.13127 PMC5328770 28242819

[17] Yang HJ, Kim MJ, Kwon DY, Kang ES, Kang S, Park S (2017). Gastroprotective actions of Taraxacum coreanum Nakai water extracts in ethanol-induced rat models of acute and chronic gastritis. J Ethnopharmacol.

[18] Kankuri E, Asmawi MZ, Korpela R, Vapaatalo H, Moilanen E (1999). Induction of iNOS in a rat model of acute colitis. Inflammation.

[19] Yang HJ, Kim MJ, Kwon DY, Kang ES, Kang S, Park S (2017). Gastroprotective actions of Taraxacum coreanum Nakai water extracts in ethanol-induced rat models of acute and chronic gastritis. Journal of ethnopharmacology.

[20] Cayan S, Coşkun B, Bozlu M, Acar D, Akbay E, Ulusoy E (2003). Botulinum toxin type A may improve bladder function in a rat chemical cystitis model. Urological research.

[21] Ma C, Donnelly DF, LaMotte RH (2010). In vivo visualization and functional characterization of primary somatic neurons. J Neurosci Methods.

[22] Chen Z, Wang T, Fang Y, Luo D, Anderson M, Huang Q (2019). Adjacent intact nociceptive neurons drive the acute outburst of pain following peripheral axotomy. Sci Rep.

[23] Lee S, Yang G, Xiang W, Bushman W (2016). Retrograde double-labeling demonstrates convergent afferent innervation of the prostate and bladder. Prostate.

[24] Pinter E, Szolcsanyi J (1995). Plasma extravasation in the skin and pelvic organs evoked by antidromic stimulation of the lumbosacral dorsal roots of the rat. Neuroscience.

[25] Verne GN, Robinson ME, Vase L, Price DD (2003). Reversal of visceral and cutaneous hyperalgesia by local rectal anesthesia in irritable bowel syndrome (IBS) patients. Pain.

[26] Li C, Xi Y, Li S, Zhao Q, Cheng W, Wang Z (2015). Berberine ameliorates TNBS induced colitis by inhibiting inflammatory responses and Th1/Th17 differentiation. Mol Immunol.

[27] Luo X, Yu Z, Deng C, Zhang J, Ren G, Sun A (2017). Baicalein ameliorates TNBS-induced colitis by suppressing TLR4/MyD88 signaling cascade and NLRP3 inflammasome activation in mice. Sci Rep.

[28] Clement CI, Keay KA, Podzebenko K, Gordon BD, Bandler R (2000). Spinal sources of noxious visceral and noxious deep somatic afferent drive onto the ventrolateral periaqueductal gray of the rat. J Comp Neurol.

[29] Berkley KJ (2005). A life of pelvic pain. Physiol Behav.

[30] Amir R, Devor M (1996). Chemically mediated cross-excitation in rat dorsal root ganglia. J Neurosci.

[31] Amir R, Devor M (2000). Functional cross-excitation between afferent A- and C-neurons in dorsal root ganglia. Neuroscience.

[32] Kim YS, Anderson M, Park K, Zheng Q, Agarwal A, Gong C (2016). Coupled activation of primary sensory neurons contributes to chronic pain. Neuron.

[33] Willis WD (1999). Dorsal root potentials and dorsal root reflexes: a double-edged sword. Exp Brain Res.

[34] Arcourt A, Gorham L, Dhandapani R, Prato V, Taberner FJ, Wende H (2017). Touch receptor-derived sensory information alleviates acute pain signaling and fine-tunes nociceptive reflex coordination. Neuron.

[35] Choi JC, Kim J, Kang E, Lee JM, Cha J, Kim YJ (2016). Brain mechanisms of pain relief by transcutaneous electrical nerve stimulation: A functional magnetic resonance imaging study. Eur J Pain.

